# Correlates of bike share use and its association with weight status at an urban university

**DOI:** 10.1371/journal.pone.0270870

**Published:** 2022-08-03

**Authors:** Lawrence H. Stahley, Sarah M. Camhi, Julie A. Wright, Philip J. Troped

**Affiliations:** 1 Department of Exercise and Health Sciences, University of Massachusetts Boston, Boston, Massachusetts, United States of America; 2 Department of Kinesiology, University of San Francisco, San Francisco, California, United States of America; Universitat de Valencia, SPAIN

## Abstract

**Background:**

Research on the influences on bike share use and potential favorable relationships between use and obesity is limited, particularly in the U.S. context. Therefore, the aims of this exploratory study were to examine correlates of awareness and use of Boston’s Bluebikes bike share system and assess the association between use and weight status.

**Methods:**

Students, faculty, and staff (n = 256) at a public urban university completed an online survey that assessed sociodemographic, behavioral, and physical activity characteristics, Bluebikes awareness, and use of Bluebikes and personal bikes. Multivariable logistic regression models were estimated to examine associations between sociodemographic and behavioral factors and bike share awareness and use; and between use and overweight/obesity status.

**Results:**

Respondents were mostly students (72.2%), female (69.1%), White (62.1%), and the mean age was 32.4±13.8 years. The percentage of respondents classified as aware of Bluebikes was 33.6% with only 12.9% reporting any use of the system. Living in a community where bike share stations were located (odds ratio (OR) = 2.01, 95% confidence interval (CI): 1.10, 3.67), personal bike ownership (OR = 2.27, 95% CI:1.27, 4.45), and not exclusively commuting to campus via car (OR = 3.19, 95% CI:1.63, 6.22) had significant positive associations with awareness. Living in a bike share community (OR = 2.34; 95% CI:1.04, 5.27) and personal bike ownership (OR = 3.09; 95% CI:1.27, 7.52) were positively associated with bike share use. Any reported use of Bluebikes was associated with 60% lower odds of being overweight/obese (OR = 0.40; 95% CI:0.17, 0.93).

**Conclusions:**

Several environmental and behavioral variables, including access to stations and personal bicycle ownership, were significantly associated with Bluebikes awareness and use. Findings also suggest a potential benefit to bike share users in terms of maintaining a healthy weight, though further longitudinal studies are needed to rule out the possibility that more active and leaner individuals tend to use bike share more frequently.

## Introduction

Despite the importance of PA for the prevention and control of obesity and other chronic diseases and conditions, less than 5% of adults and adolescents in the United States (U.S.) are meeting guidelines for moderate-to-vigorous activity according to accelerometer-based surveillance data [1]. Historically, the focus for physical activity (PA) promotion efforts within the U.S. has been on leisure time activity. However, recent national public health objectives, such as those identified in Healthy People 2030, also emphasize engagement in PA for travel to and from destinations; in other words, active transportation [2]. Participation in one form of active transportation, utilitarian cycling, may have the potential to increase overall adherence rates to PA guidelines. For example, a study in Portland, Oregon, found that almost 60% of commuter cyclists were meeting weekly recommendations for moderate-intensity PA through utilitarian cycling [3].

A number of studies have provided a strong health-related rationale for promoting active transportation, including utilitarian cycling [4–12]. A survey of adults in 17 countries found that the lowest rates of obesity were generally found in countries with the highest rates of active transportation [6]. Among the countries included in this study the U.S. had the lowest levels of active transportation and the highest rates of obesity. Other studies have shown that walking or cycling to work or school is associated with lower levels of triglycerides, blood pressure, and insulin, higher levels of aerobic fitness, and reduced rates of obesity and cardiovascular disease [4–6, 13]. A recent analysis of state-level aggregate data in the U.S. showed statistically significant negative correlations between active commuting (defined as walking, cycling, or using public transit) and the prevalence of coronary heart disease, myocardial infarction, and stroke [12]. Studies specifically focused on utilitarian cycling have also reported health benefits. A systematic review from the Netherlands estimated that individuals switching from driving to utilitarian cycling could experience between 3 and 14 months of life gained, when the authors evaluated the potential health benefits of PA compared to the risks of cycling in an urban environment [14]. Findings from two prospective cohort studies in the United Kingdom [10] and China [11] found that adults who commuted to work by cycling had a significantly reduced risk of all-cause mortality, cardiovascular disease incidence and mortality, and cancer incidence and mortality [10], and incident ischemic heart disease and stroke [11]. Despite evidence for the health benefits of active transportation and utilitarian cycling, few studies have specifically focused on the potential health benefits of public bike share systems.

One approach for increasing rates of utilitarian cycling is through the development and promotion of bike share. Public bike share systems provide bicycles to rent for a small fee; bikes are picked up and returned to any docking station in the system or in the case of dockless bike share systems, bicycles can be parked within defined areas at bike racks or along sidewalks. Public bike share has operated in Europe for decades. However, the first modern bike share programs in the U.S. opened in 2007 in Tulsa, Oklahoma, and in 2008 in Denver, Colorado [15]. From 2010 to 2016 the number of publicly-available bike share systems, defined as those with at least 10 stations and 100 bikes, increased from 4 to 55 systems [16]. In 2019 there were 40 million trips on station-based bike share systems in the U.S., which included both pedal and e-bikes (uses an electric motor that can be used to assist with or replace pedaling), and 10 million dockless e-bike share trips [17]. Although the number of U.S. bike share systems has increased rapidly, few North American studies have examined the factors associated with awareness or use of bike share, or the association between bike share use and health-related outcomes such as weight status. In addition, there are few U.S. studies of bike share focused on the university context [18–20]. In one randomized controlled pilot study with 56 college students in the Kansas City area, researchers found limited effects of a one-month free bike share membership on bicycling activity [18]. A second study examined bike share use at stations located at 25 universities and found that ridership was higher at private versus public universities, on campuses where Latino and Asian students were the dominant ethnic groups, on campuses closer to the central business district, and when daily temperature was higher and there were less severe weather events [19]. None of these studies examined effects of bike share on weight status or health-related outcomes.

Evidence on the correlates of bike share awareness and use is critical for designing effective interventions, including transportation and public health policies aimed at promoting bike share use. Some studies have shown that bike share users tend to be younger (18–24 years old), White, male, and have higher education levels and employment rates [21, 22], suggesting the need for greater efforts to promote bike share among older, racially diverse, and lower income groups. Earlier research from North America showed the most common purpose for using bike share programs was to travel to and from work or school, with a majority of trips being part of a multimodal commute that included some form of public transportation [21–23]. However, a recent examination of correlates of frequent bike share use in the U.S. found that a slightly higher percentage of users identified social/entertainment (18%) and exercise (21%) as the reason for using bike share compared to commuting only (15%) [24].

A well-established environmental correlate of bike share use is living in proximity to a docking station [22, 25]. For example, research on the BIXI bike share program in Montreal, Canada showed that living within 250 meters of a bike share station significantly increased the likelihood of using bike share [25]. Two recent studies have examined correlates of dockless bike share use [20] and access [26]. An investigation of dockless bike share at a Texas university found that living off campus, lower class rank, current biking behavior, and confidence in biking were all positively correlated with bike share use [20]. Mooney and colleagues assessed spatial access to dockless bike share bikes during the first six months of a pilot program in Seattle and found that neighborhoods with above the median bike availability had higher median incomes, more college-educated residents, and more access to opportunity on average [26]. Despite the rapid growth in dockless bike share and scooter systems in the U.S. since 2017, station-based bike share still comprised 29% of all trips in 2019 [17]. Six major urban markets that included Boston, New York, Washington D.C., Chicago, the Bay Area, and Honolulu generated 87% of dock-based trips in that year [17].

There is also limited research on bike share’s potential for increasing physical activity and thereby having a positive effect on health-related outcomes, such as obesity. Researchers in Minneapolis, MN found that bike share users in low-income communities had an average trip duration of 22 minutes or approximately two-thirds of the recommended daily physical activity [27]. A simulation study from Barcelona, Spain, estimated that an increase in bike share use could reduce approximately 12 deaths a year through increased PA alone, while also lowering carbon dioxide emissions in the city from decreased car usage [28]. Two U.S. based studies have focused on bike share and weight status. In a cross-sectional study of bike share users in Washington, DC, participants reported losing weight and lowering their stress levels after using the local system [29]. Xu conducted a county-level analysis of obesity rates before and after the introduction of bike share programs compared to rates in counties without bike share and found on average 0.5% to 1.0% reductions in obesity prevalence [30].

Given the limited evidence on correlates of awareness and bike share use, and health benefits of bike share systems within the U.S. context, this exploratory survey study was designed to focus on Bluebikes, a bike share system in Boston, MA, and surrounding communities. Bluebikes was launched in July 2011 with 60 stations and 610 bicycles and by the end of 2019 had expanded to 325 stations and over 3500 bicycles [31]. Bluebikes trips increased from 140,974 in 2011 to 1,184,558 trips in 2014 and as of 2019 had doubled to approximately 2.5 million trips [32].

Despite the Bluebikes system’s growth, little is known about the levels of awareness and use of Bluebikes or the potential health benefits to users. Therefore, the aims of this study were to: 1) examine the correlates of awareness and use of Bluebikes among students, faculty, and staff at a public urban university in Boston; and 2) examine associations between bike share use and overweight/obesity status.

## Materials and methods

### Study design

A cross-sectional survey was conducted to assess awareness and utilization of the Bluebikes bike share system among students, faculty, and staff at an urban public university during the fall of 2014. At the time of data collection, the university had over 16,000 students, 59% were female and 41% male. Fifty-six percent of students were White, 16% were Black/African American, 12% were Asian, and 12% were Hispanic. Sixty-seven percent of faculty and 60% of staff were White. Since there was no on-campus housing, all students commuted to campus. This university provided an opportunity to study a racially and ethnically diverse population with close access to two docking stations. One station was located at the public train and bus station approximately one mile from campus. A second Bluebikes station was located across the street from the university campus center.

### Participants and recruitment

The target population for this study consisted of students, faculty, and staff of the university. Inclusion criteria were: 1) has a valid university email address, 2) can read and comprehend English, 3) willing and able to give informed consent. There were no restrictions based on age, sex, race/ethnicity, current PA levels, or prior knowledge or use of Bluebikes. Two primary methods of recruitment were utilized: 1) multiple e-mail contacts; and 2) face-to-face contact in the campus center. Two emails (initial and follow-up) were sent to all faculty (n~ 1000) and both undergraduate and graduate students in the nursing and health sciences college at the university (n~1147) requesting their participation in the study. The email included a brief description of the study and a link to the online survey. A follow-up email was sent about one week after the initial email to increase the response rate.

Face-to-face recruitment was also conducted for two full days during the fall semester in the university’s campus center. Students, faculty, and staff who met inclusion criteria were given the option to take the online survey immediately on a laptop computer or personal phone, or provide their email address and be sent a link to the survey. Recruitment began in October 2014 and continued until the survey was closed in December 2014. When participants clicked on the link to the survey, they were brought to an informed consent page. Continued participation after reading this page indicated the participant’s consent. Individuals who completed the survey were able to enter a drawing for a $25 gift card. This study was approved by the University of Massachusetts Boston’s Institutional Review Board.

At the end of the data collection phase, there were 301 surveys submitted online. After checking for data completeness, 45 (15%) surveys were excluded from the analysis due to missing responses for key independent or dependent variables, leaving a final analytic sample of 256 individuals. Among this sample there were 185 students (33 graduate-level) and 71 faculty/staff.

### Survey

The online survey was created using the SNAP Survey Software (Snap Surveys, Portsmouth, NH). The survey consisted of 44 questions that assessed: 1) sociodemographic characteristics, 2) awareness and utilization of Bluebikes, 3) routine PA and commuting patterns, 4) facilitators and barriers to bike share use, 5) bicycle helmet use, 6) bicycle accidents, and 7) height and weight. New survey items were developed, as well as adapted from previous physical activity and bike share surveys [33–35].

### Dependent variables

Dichotomous outcomes were created for both awareness and utilization of Bluebikes. Awareness was defined as having answered “yes” to knowing about Bluebikes and correctly reporting the locations of the two Bluebikes docking stations near the university. Use was defined as having used Bluebikes at any time in the past (yes/no) at any location throughout the bike share system. Respondents self-reported their height and weight, which was used to calculate body mass index (BMI) (kg/m^2^). A dichotomous outcome was created, combining overweight and obese individuals into one group (BMI ≥ 25.0 kg/m^2^), while BMI < 25.0 kg/m^2^ was classified as underweight/normal weight [36].

### Independent variables

Sociodemographic and behavioral variables were examined as potential correlates of Bluebikes awareness and use. Sociodemographic variables included age, sex, race, Hispanic/Latino ethnicity, location of home residence, and university affiliation (i.e., student or faculty/staff). Respondents were asked to provide their home zip code to determine whether they lived in any of the four cities/towns where bike share stations were located: Boston, Cambridge, Somerville, or Brookline.

Behavioral variables included the mode of commuting to campus, frequency of PA per week, and whether the respondent owned a bicycle (yes/no). Individuals who exclusively drove a vehicle to campus were compared to those who used other forms of commuting that included public transportation, walking, and cycling. PA was assessed with the question, “In the past week, on how many days have you done a total of 30 minutes or more of physical activity, which was enough to raise your breathing rate? This may include sport, exercise and brisk walking or cycling for recreation or to get to and from places but should not include housework or physical activity that may be part of your job” [35].

### Statistical analysis

All statistical analyses were conducted with SAS, Version 9.4 software (Copyright © 2015 SAS Institute). Univariate statistics (e.g., means, frequencies) were used to summarize all variables. Multiple logistic regression was used to assess associations between sociodemographic and behavioral variables and both awareness and use of Bluebikes. Logistic regression was also used to examine the association between participants’ bike share use and the odds of being overweight/obese. Three regression models were estimated for each bike share outcome, sequentially controlling for: 1) age, 2) additional sociodemographic variables (sex, race, Hispanic ethnicity, university affiliation (student, faculty, staff), and living/not living in an area with Bluebikes), and 3) both sociodemographic and behavioral variables (mode of commuting, frequency of physical activity, and bike ownership).

## Results

### Demographic characteristics of respondents

Demographic characteristics for the overall sample (N = 256) and participants stratified by university affiliation are reported in Table 1. The average age of all participants was 32.4 years, with students making up 72% (n = 185) of the overall sample. Thirty-three respondents were graduate students. Approximately 69% of participants were female and 62% were White. Approximately 8% of respondents were Hispanic/Latino and about 39% of participants lived in a community with access to a Bluebikes station.

**Table 1:** Sociodemographic, commuting, and behavioral characteristics of survey respondents at a public urban university.

	Overall N = 256	Students n = 185	Faculty/staff n = 71
**Sex % (n)**			
**Male**	30.9 (79)	25.4 (47)	45.1 (32)
**Female**	69.1 (177)	74.6 (138)	54.9 (39)
**Age in years (SD)**	32.4 (13.8)	26.4 (8.3)	48.0 (13.0)
**Race % (n)**			
**White**	62.1 (159)	54.1 (100)	83.1 (59)
**African American/Black**	13.3 (34)	17.3 (32)	2.8 (2)
**Asian**	10.6 (27)	11.4 (21)	8.5 (6)
**Other**^**a**^	14.1 (36)	17.3 (32)	5.6 (4)
**Hispanic % (n)**	8.2 (21)	10.3 (19)	2.8 (2)
**Lives in Bluebikes area % (n)**	39.1(100)	37.8 (70)	42.3 (30)
**Mode of commuting %(n)**			
**Drive**	59.8 (153)	63.2 (117)	50.7(36)
**Public transport**	48.8 (125)	43.2 (80)	63.4 (45)
**Walk**	22.3 (57)	24.3 (45)	16.9 (12)
**Cycle**	7.4 (19)	2.2 (4)	21.1(15)
**Weight status**^**b**^ **% (n)**			
**Underweight/healthy weight**	57.4 (147)	57.8 (107)	56.3 (40)
**Overweight/obese**	42.6 (109)	42.2 (78)	43.7 (31)
**PA days/week (SD)**	3.2 (2.1)	3.1 (2.1)	3.4 (1.8)
**Owns a bicycle % (n)**	52.0 (133)	44.3 (82)	71.8 (51)
**Aware of Bluebikes and station locations % (n)**			
**Yes**	33.6 (86)	27.0 (50)	50.7 (36)
**No**	66.4 (170)	73.0 (135)	49.3 (35)
**Ever used Bluebikes**^**c**^ **% (n)**			
**Yes**	12.9 (33)	10.8 (20)	18.3 (13)
**No**	87.1 (223)	89.2 (165)	81.7 (58)

^a^ “Other” includes individuals self-identifying as Native Alaskan, Pacific Islander, or from multiple races.

^b^ Weight status: underweight/healthy weight = BMI < 25.0; overweight/obese = BMI ≥ 25.0.

### Commuting and physical activity characteristics

The most common mode of commuting to and from campus was driving, with almost 60% of respondents reporting at least some driving as part of their typical commute. Public transportation (48.8%) was the second most common mode of travel. Less than 30% of respondents reported incorporating walking or cycling into their commute to or from campus.

On average, both students and faculty/staff reported that they were physically active about three days per week. Almost 26% of participants were overweight and 16.4% were obese. Approximately 52% of respondents reported owning a personal bicycle, and in the past year, these respondents took an average of 55.5 bicycle trips.

### Facilitators and barriers to bike share use

Participants reporting Bluebikes use at any time in the past were asked why they used the program. The three most common reasons were for recreation/leisure (60.6%), running errands (33.3%), and getting to work (27.2%). Individuals who had never used the system were asked why they did not use Bluebikes, and the most common responses were fear (40.5%), lack of interest (33.1%), station availability (32.4%), cost (25.7%), and use of their own bike (24.3%).

### Correlates of bike share awareness

Correlates of Bluebikes awareness are shown in Table 2. In age-adjusted analyses, living in a Bluebikes community, faculty, or staff affiliation with the university, not exclusively commuting via car, and owning a personal bicycle all showed positive, statistically significant associations with awareness. In a multivariable model that included all demographic and behavioral variables, three of these variables remained statistically significant: living in an area with Bluebikes stations (OR = 2.01; 95% CI:1.10, 3.67), not exclusively commuting via car (OR = 3.19; 95% CI:1.63, 6.22), and owning a bicycle (OR = 2.37; 95% CI:1.27, 4.45). Individuals living in one of the communities where Bluebikes operates were about two times more likely to be aware of Bluebikes than those living in other communities. Respondents who used public transportation, walked, or cycled as part of their commute to campus were over three times more likely to be aware of Bluebikes than those who reported only driving. Bicycle owners were about two times as likely to be aware of Bluebikes, compared to those who did not own a personal bike.

**Table 2:** Demographic and behavioral correlates of bluebikes awareness (n = 256).

	Age-adjusted models			Demographic model			Demographic and behavioral model		
	OR^a^	95% CI^b^		OR	95% CI		OR	95% CI	
**Age**				1.00	0.97	1.03	1.00	0.98	1.04
**Sex**									
**Female**	1.00			1.00			1.00		
**Male**	0.85	0.47	1.51	0.79	0.42	1.46	0.67	0.35	1.29
**Race**									
**White**	1.00			1.00			1.00		
**Minority**	0.73	0.41	1.29	0.81	0.44	1.48	0.81	0.43	1.55
**Hispanic**									
**No**	1.00			1.00			1.00		
**Yes**	1.75	0.70	4.38	2.03	0.79	5.24	2.27	0.84	6.10
**University affiliation**									
**Student**	1.00			1.00			1.00		
**Faculty/staff**	**3.54**	**1.55**	**8.06**	**3.16**	**1.34**	**7.45**	2.19	0.89	5.35
**Lives in Bluebikes area**									
**No**	1.00			1.00			1.00		
**Yes**	**2.46**	**1.43**	**4.23**	**2.27**	**1.30**	**3.96**	**2.01**	**1.10**	**3.67**
**Mode of commuting**									
**Drives only**	1.00						1.00		
**Other modes**	**3.51**	**1.94**	**6.36**				**3.19**	**1.63**	**6.22**
**Frequency of PA/week**	1.05	0.92	1.19				0.94	0.81	1.09
**Owns personal bike**									
**No**	1.00						1.00		
**Yes**	**1.93**	**1.11**	**3.33**				**2.37**	**1.27**	**4.45**

^a^ OR = odds ratio

^b^ 95% CI = 95% confidence interval

Notes on models: Each age-adjusted model included 1 independent variable and age.

The one demographic model included all 6 variables listed.

The one demographic/behavioral model included the 6 demographic and 3 behavioral variables.

### Correlates of bike share use

In age-adjusted and multivariable models, living in a Bluebikes community and owning a bike had statistically significant positive associations with use of Bluebikes (see Table 3). No other sociodemographic or behavioral variables were associated with bike share use. Personal bike owners were over three times more likely to have used Bluebikes in the past than non- owners (OR = 3.09; 95% CI: 1.27, 7.52). Participants living in a Bluebikes community (i.e., Boston, Brookline, Cambridge, and Somerville) were more than two times as likely to have used the system, compared to those living outside these communities (OR = 2.34; 95% CI: 1.04, 5.27).

**Table 3:** Demographic and behavioral correlates of bluebikes use (n = 256).

	Age adjusted models			Demographic model			Demographic and behavioral model		
	OR^a^	95% CI^b^		OR	95% CI		OR	95% CI	
**Age**				1.00	0.97	1.04	1.00	0.96	1.04
**Sex**									
**Female**	1.00			1.00			1.00		
**Male**	0.89	0.39	2.01	0.87	0.38	2.00	0.78	0.33	1.87
**Race**									
**White**	1.00			1.00			1.00		
**Minority**	0.87	0.39	1.93	0.94	0.41	2.15	1.17	0.48	2.83
**Hispanic**									
**No**	1.00			1.00			1.00		
**Yes**	1.24	0.34	4.54	1.34	0.36	5.00	1.28	0.33	4.96
**University affiliation**									
**Student**	1.00			1.00			1.00		
**Faculty/staff**	1.98	0.67	5.85	1.71	0.56	5.21	1.46	0.47	4.61
**Lives in Bluebikes area**									
**No**	1.00			1.00			1.00		
**Yes**	**2.16**	**1.03**	**4.56**	2.05	0.96	4.35	**2.34**	**1.04**	**5.27**
**Mode of commuting**									
**Drives only**	1.00						1.00		
**Other modes**	1.36	0.63	2.91				1.04	0.44	2.47
**Frequency of PA/week**	1.07	0.89	1.20				1.00	0.82	1.22
**Owns personal bike**									
**No**	1.00						1.00		
**Yes**	**2.69**	**1.18**	**6.18**				**3.09**	**1.27**	**7.52**

^a^ OR = odds ratio

^b^ 95% CI = 95% confidence interval

Notes on models: Each age-adjusted model included 1 independent variable and age.

The one demographic model included all 6 variables listed.

The one demographic/behavioral model included the 6 demographic and 3 behavioral variables.

### Association between bike share use and overweight/obesity

Bluebikes use had a statistically significant negative association with overweight/obesity, after controlling for potential confounding by age, sex, race, university affiliation, living in a Bluebikes area, and frequency of engaging in physical activity. Survey respondents who reported any Bluebikes use had a 60% lower odds of being overweight/obese, as compared to nonusers (OR = 0.40; 95% CI = 0.17, 0.93). There was no association between personal bike ownership and weight status (OR = 0.98, 95% CI = 0.57, 1.69).

## Discussion

This study of students, faculty, and staff at an urban public university in Boston found significant positive associations between use of Bluebikes and living in a community with access to Bluebikes docking stations and personal bike ownership. These two variables, as well as commuting to campus via public transportation, walking, or cycling, were also found to have significant positive associations with awareness of this bike share system. In addition, bike share use had a statistically significant negative association with overweight/obesity status.

In multivariable models, no sociodemographic variables were associated with either awareness or use of Bluebikes. These findings are somewhat inconsistent with previous studies, which have found bike share users tend to be younger, White, male, and have higher socioeconomic status [21, 22]. A recent study of U.S. bike share users found that gender was not a predictor of more frequent versus less frequent use, income was a significant correlate for those who used bike share for social/entertainment purposes, and African-American and Hispanic users were more likely to make more frequent trips than Caucasians depending on the trip purpose [24]. Another study of bike share at 25 U.S. universities found that campuses with Latino and Asian as the two dominant ethnic groups had the highest bike share ridership, as compared to the combinations of other ethnic groups [19]. Even though two-thirds of Bluebikes users in the present study were White, race was not associated with use. It is not clear why sex and age were not associated with either awareness or use, however, a lack of variability in the sample may have contributed to the null findings. Income and socioeconomic status were not assessed in this study. The lack of associations for demographic factors may not be generalizable to other public or private urban universities in the Boston area and elsewhere in the U.S., due to the unique demographic and geographic characteristics of this university. At the time of this study there was no on-campus housing and 53% of female and 51% of male undergraduates were U.S. students of color [37].

Respondents who lived in a community where Bluebikes operated (i.e., Boston, Brookline, Cambridge, or Somerville) were significantly more likely to be aware of Bluebikes and use it than individuals living outside of these areas. These results are not unexpected since those residing in areas where Bluebikes operates would have more opportunities to be exposed to stations and use the system. These results are generally consistent with findings from studies of other North American bike share systems, which have shown that users tend to either live within an inner urban area, within 250-m of a docking station, or closer to work than nonusers [23, 38, 39].

Owning a bike was also positively associated with awareness and use of Bluebikes. This finding is similar to results from several other studies which found that members of bike share systems were more likely to own and use personal bikes than nonmembers [23, 40, 41]. Since bike owners already have interest and experience with cycling, they are likely to have more confidence in their cycling abilities and may be more aware of current cycling-related programs and news. Kellstedt and colleagues found that confidence in cycling was a significant positive correlate of bike share use in a dockless system at a large Texas university [20]. Prior experience and confidence may be especially important for engaging in cycling in urban settings where there is more traffic and potential conflicts with drivers. In fact, the present study found that the most common reason for not using Bluebikes was fear, which was cited by 40.5% of nonusers. This is consistent with previous literature indicating that safety concerns are a major barrier for bike share participation, commuter cycling, and active transportation in general [41–45]. For example, in an Australian study researchers determined that positive attitudes towards cycling and perceived behavioral control increased the odds of cycling for both transport and recreation [45]. It appears that an individual’s perceived ability to cycle, and specifically to be safe, is a critical factor to address if bike share systems are to gain more widespread adoption. Currently the evidence on the necessity of having certain built environment infrastructure such as bike lanes to enhance safety and support ridership appears mixed. For example, an investigation of Hamilton, Ontario’s bike share system found that proximity to important locations in the city had a strong relationship with ridership, yet the amount of bike lanes within a 200-meter buffer around bike share stations had little to no association with ridership [46]. Gender may play a role in the associations between cycling infrastructure, cyclists’ confidence and safety perceptions, and ridership. A study of New York’s Citi Bike system showed that the length of off-street bicycle routes, the number of benches and bicycle racks (for personal bikes), intersection density, and green space all had significant positive associations with the proportion of bike share trips made by women [47].

Since bike share systems have the potential to increase the number of individuals who actively commute, it is important to understand how different modes of commuting are associated with bike share awareness and use. In one study that assessed bike share systems around the globe, researchers found that a majority of bike share trips included some form of public transportation [21]. Given that one Bluebikes docking station is located at a public transportation station (bus and subway) within a mile of the university, there is potential for Bluebikes to be used as part of a multimodal active commute to the campus. Although over 70% of participants reported having heard of the term Bluebikes, only about a third reported being both aware of Bluebikes and correctly identified the locations of the two docking stations near campus. This suggests that additional promotional efforts, including signs directing people towards bike share stations may be beneficial. These efforts should also emphasize that active transportation and specifically bike share use can be a healthy alternative to driving that may lead to significant health benefits including lower rates of overweight/obesity.

An unexpected finding in the present study was that the most common reason identified for using Bluebikes was for recreation or leisure purposes and less frequently to run errands and commute to work. This indicates that bike share systems such as Bluebikes should not be solely viewed through an active transportation lens, but also as a low-cost resource for leisure-time physical activity. The multiple purposes for using bike share was also seen in a recent study of 1711 U.S. bike share users in which the investigators grouped participants into 5 groups based on trip purpose; 15% for commuting only, 18% for social/entertainment, 21% for exercise, 29% for multiple purposes, and 17% for “other” [24]. Chen and Hsu also found differences in correlates of use by purpose. An implication of the trip purpose results from these two studies is that promotional strategies probably need to be tailored to potential users’ primary motivation for using bike share [24]. As noted, use of the Bluebikes bike share system was associated with a significantly reduced odds of being overweight/obese. To our knowledge this is one of the first studies in the U.S. context to specifically document an inverse relationship between bike share use and weight status. As previously mentioned, in a recent 2019 analysis of county-level obesity rates, Xu estimated that the introduction of bike share systems resulted in small improvements in obesity prevalence [30]. Also, as noted earlier, an international study on active transportation and weight status found that countries with the highest rates of active transportation tended to have the lowest rates of obesity, although this research did not specifically focus on bike share use [6]. Similarly, a comprehensive analysis of city and state data in the U.S., and international data consistently showed that higher levels of walking and cycling to work were associated with lower obesity and diabetes rates and higher rates of meeting PA guidelines [48]. Collectively, findings from the present study and previous research indicates the potential for utilitarian cycling and bike share use to positively influence overweight/obesity rates.

A strength of this study is that it contributes to a limited literature on correlates of bike share awareness in the U.S. context, as well as demonstrates an association between bike share use and weight status. Another strength was that participants were part of a racially and ethnically diverse urban campus where all students and faculty lived off campus. Also, the racial and ethnic composition of the students in the study was comparable to that of the overall student population at the university. African American/Black and Hispanic faculty and staff may be underrepresented. With a large proportion of commuters driving or using public transportation, there appears to be potential for a greater percentage of students, faculty, and staff at this university to become active commuters and incorporate bike share use into trips to or from campus.

This study also has several limitations. The cross-sectional design does not allow us to determine the direction of the relationship between bike share use and weight status. This design prevents us from determining whether bike share use leads to improvements in weight status or whether leaner and more physically fit individuals are choosing to use bike share more. Future prospective studies that can assess weight status before and after individuals start to use bike share are needed. Another limitation is the self-report measures of bike share use, which have not been tested for reliability and validity. The use of a convenience sample and the low overall response rate were also limitations. The recruitment method of mass emailing to students in the college of nursing and health sciences may have resulted in bias in the sample with a higher representation of health-conscious students as compared to the overall university population. It is possible that the associations we observed between living in a community where Bluebikes operated and bike share use were confounded by neighborhood-level socio-demographic factors.

As noted previously the U.S. landscape for micromobility options has changed dramatically in the past 4 to 5 years with growth in dockless systems, e-bikes, and scooters. A final limitation of this study is that the findings cannot be assumed to pertain to these other technologies. More research is needed to directly compare correlates of use for all these micromobility options and the potential health benefits of each technology. This type of comparative research is important given that dock-based systems continue to have a strong presence in large U.S. markets such as Boston.

### Conclusions

The use of the Bluebikes bike share system was associated with a decreased odds of being overweight/obese among students, faculty, and staff at an urban public university. Bike share use appears to have the potential to influence health related outcomes such as overweight/obesity rates though evidence from prospective studies is needed before firm conclusions can be made. On this urban campus awareness and use of bike share were fairly low, indicating an opportunity for bike share systems and universities to work together and develop creative approaches to more effectively promote bike share as healthy alternatives to driving or as part of a multimodal trip to and from campus, or as an opportunity to integrate leisure time physical activity into time on campus. As the findings from this study and others indicate, it appears that a combination of locating bike share stations close to transportation hubs and key destinations, as well as sustained promotional efforts will be needed to increase awareness and use of these systems.
